# Emotional valence sensing using a wearable facial EMG device

**DOI:** 10.1038/s41598-021-85163-z

**Published:** 2021-03-11

**Authors:** Wataru Sato, Koichi Murata, Yasuyuki Uraoka, Kazuaki Shibata, Sakiko Yoshikawa, Masafumi Furuta

**Affiliations:** 1 Psychological Process Team, BZP, RIKEN, 2-2-2 Hikaridai, Seika-cho, Soraku-gun, Kyoto, 619-0288 Japan; 2 Field Science Education and Research Center, Kyoto University, Oiwake-cho, Kitashirakawa, Sakyo 606-78502 Japan; 3 Shimadzu Corporation, 3-9-4 Hikaridai, Seika-cho, Soraku-gun, Kyoto, 619-0237 Japan; 4 PROKIDAI Co.,Ltd., 3-5, Hikaridai, Seika-cho, Soraku-gun, Kyoto, 619-0289 Japan; 5 Kyoto University of the Arts, 2-116 Uryuyama Kitashirakawa, Sakyo, Kyoto, Kyoto 606-8271 Japan

**Keywords:** Human behaviour, Neurophysiology

## Abstract

Emotion sensing using physiological signals in real-life situations can be practically valuable. Previous studies have developed wearable devices that record autonomic nervous system activity, which reflects emotional arousal. However, no study determined whether emotional valence can be assessed using wearable devices. To this end, we developed a wearable device to record facial electromyography (EMG) from the corrugator supercilii (CS) and zygomatic major (ZM) muscles. To validate the device, in Experiment 1, we used a traditional wired device and our wearable device, to record participants’ facial EMG while they were viewing emotional films. Participants viewed the films again and continuously rated their recalled subjective valence during the first viewing. The facial EMG signals recorded using both wired and wearable devices showed that CS and ZM activities were, respectively, negatively and positively correlated with continuous valence ratings. In Experiment 2, we used the wearable device to record participants’ facial EMG while they were playing Wii Bowling games and assessed their cued-recall continuous valence ratings. CS and ZM activities were correlated negatively and positively, respectively, with continuous valence ratings. These data suggest the possibility that facial EMG signals recorded by a wearable device can be used to assess subjective emotional valence in future naturalistic studies.

## Introduction

The ability to use physiological signals to sense emotional states in real-life situations offers practical advantages. Emotions carry special significance, as positive and negative emotional experiences are crucial components of human subjective wellbeing^1^ and affect behavior and health^2^. The current gold standard for assessing emotional states is self-reports measures, based on discrete (e.g., anger) or dimensional (e.g., valence) perspectives^3,4^. Numerous previous studies have shown that self-report measures can reveal emotional states, allowing prediction of future behaviors (e.g., shopping behaviors^5^) and mental states (e.g., depression^6^). However, self-report measures of emotional state have several intrinsic limitations, such as their subjective nature and inclusion of biases, as well as the difficulty of continuous recording while conducting other tasks^7^. Recording physiological signals to assess subjective states can complement subjective assessments by providing objective, unbiased, and continuous data^7^. If such emotion sensing were possible not only in laboratories but also in natural environments, this method could help us understand and improve everyday life^8–10^.

Several wearable devices that can record physiological signals to assess emotional states in naturalistic situations have been developed^9,11,12^. These devices typically record autonomic nervous system activity, such as electrodermal activity (EDA). Although most commercially available wearable devices have not been empirically validated^9^, a few studies have reported that physiological recordings made with wearable devices were useful for assessing emotional responses. For example, a previous study reported that EDA recorded during grocery store shopping was associated with emotional experiences and buying behaviors^13^. Another study reported that EDA recorded during daily life detected stress^14^.

A number of previous psychophysiological studies using traditional wired devices have revealed concordance between physiological signals and dimensional subjective emotional experiences. Specifically, these studies reported that autonomic nervous system activity measures, such as EDA, consistently reflected emotional arousal, which is the energetic or quantitative dimension of emotional experience; the studies also demonstrated that facial electromyography (EMG) recorded from the corrugator supercilii (CS) and zygomatic major (ZM) muscles reflected emotional valence, which is qualitative measure of emotional experience^15,16^. For example, a previous study analyzed subjective ratings of valence and arousal, EMG data from the CS and ZM muscles, and EDA while participants observed various emotional images^17^. The results showed a positive association between arousal ratings and EDA activity and negative and positive associations with CS and ZM activities, respectively. Several other studies confirmed that facial EMG recorded from the CS and ZM muscles corresponded to subjective valence ratings obtained using images^18–20^, films^21,22^, sounds^19,23^, and words^19^. Some studies also reported that these facial EMG signals could reflect the valence of stimuli^24–26^. Because the difference in valence (e.g., fear versus excitement, both of which are associated with high arousal levels) is indispensable for assessing subjective experiences and predicting behaviors, physiological emotion sensing in real-life situations should be able to assess valence-related signals such as facial EMG. However, no validated wearable devices are available to assess emotional valence. Although a recent study developed a wearable device that can record facial EMG data from the ZM muscle^27^, no evidence was reported regarding concordance between subjective valence and facial EMG data recorded from wearable devices.

**Figure 1:**
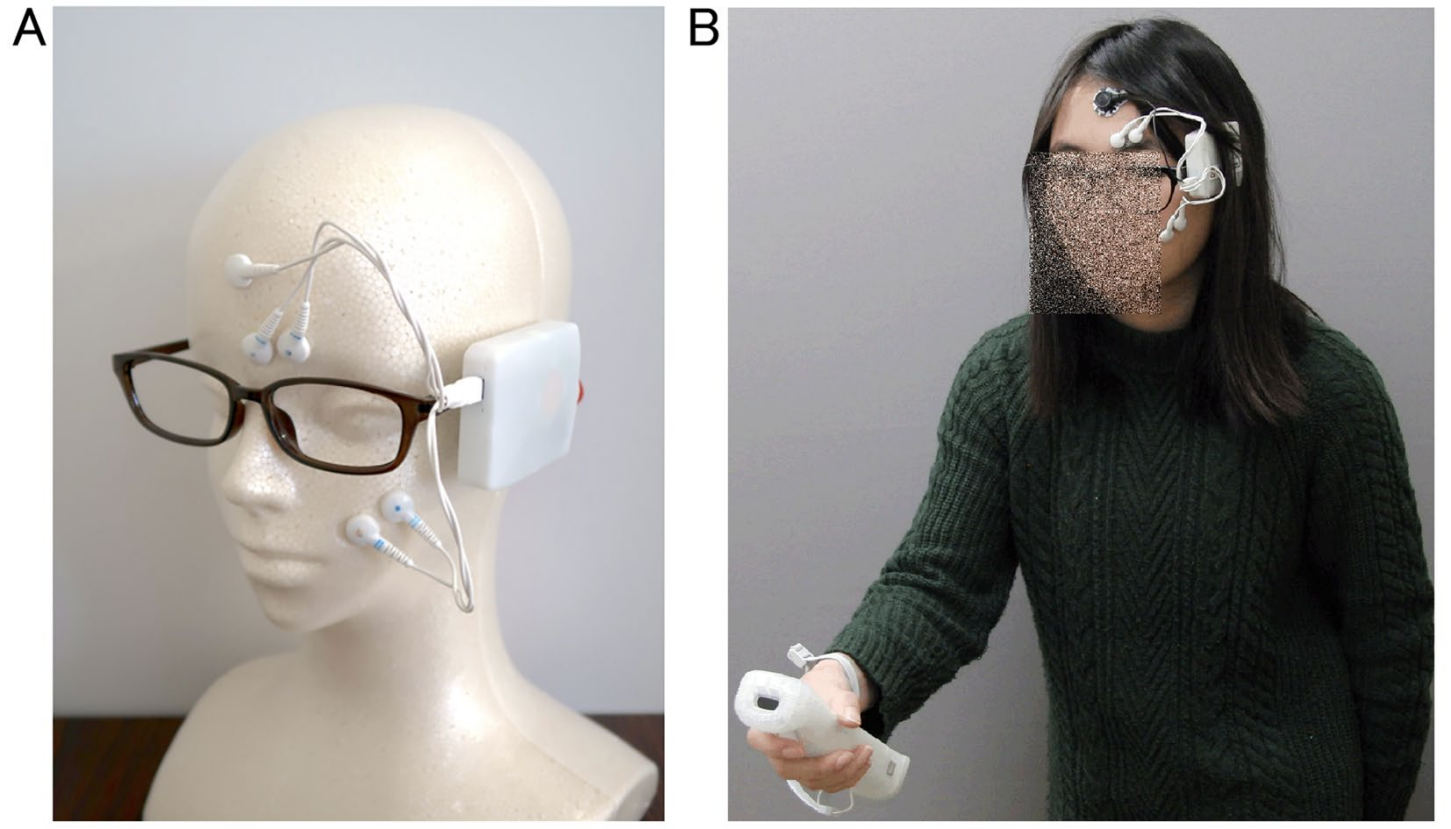
Illustrations of the wearable device (**A**) and participants using the wearable device in Experiment 2 (**B**).

**Figure 2:**
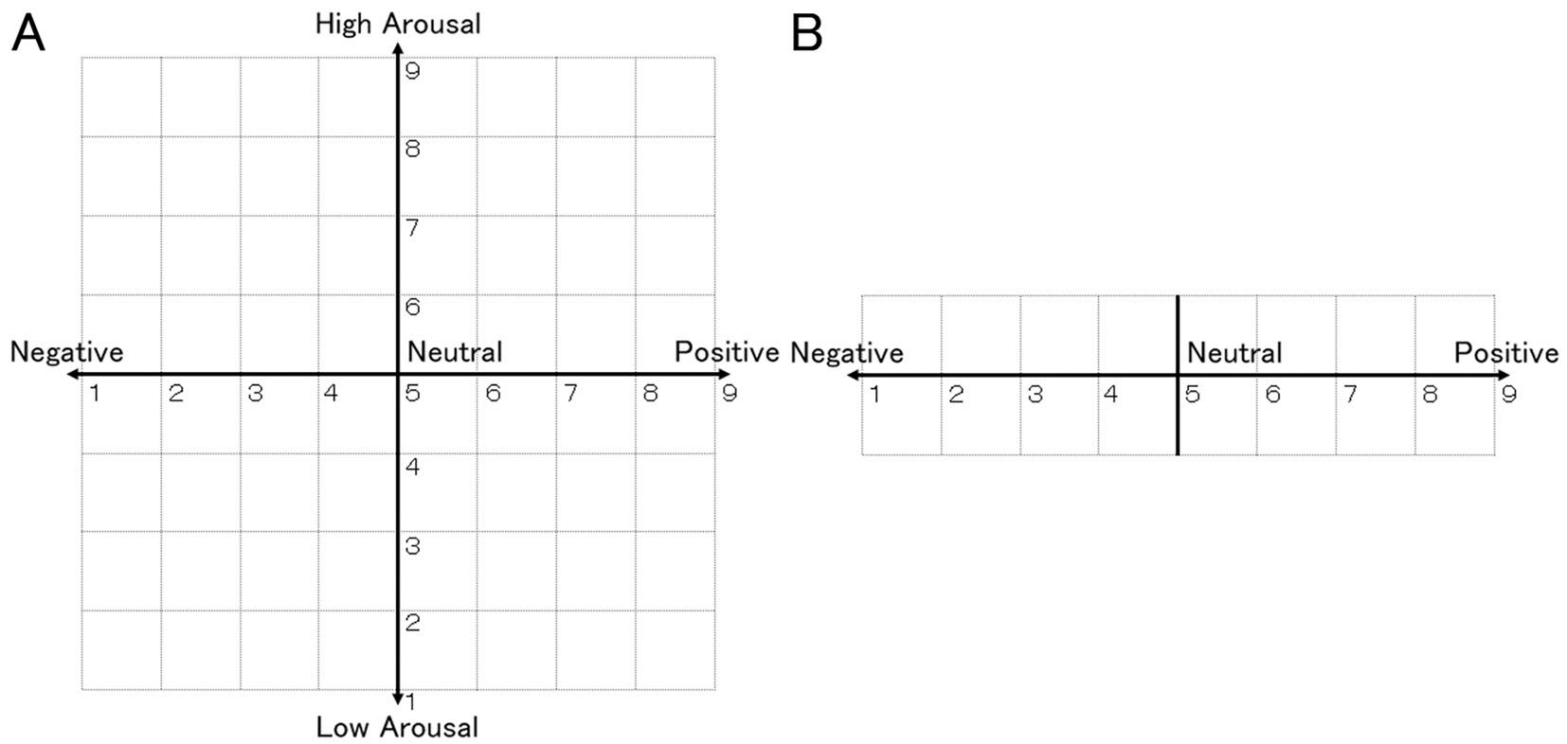
Response panels for valence and arousal ratings in Experiment 1 (**A**) and valence ratings in Experiments 1 and 2 (**B**).

To assess subjective emotional valence using physiological signals in real-life situations, we developed a wearable device to record facial EMG data from the CS and ZM muscles (Fig. 1A). The purpose of this study was to determine whether our wearable device can sense emotional valence through two laboratory experiments. In Experiment 1, we measured CS and ZM activity using wired and wearable devices while participants viewed films chosen to induce distinct emotions with valences ranging from very positive to very negative^28^. After watching each film, participants rated their overall impression in terms of valence and arousal using an affect grid^29^ (assessing the emotional state via a 9-point valence and arousal scales; Fig. 2A) to validate the stimuli. After all film clips were presented, the clips were presented again to the participants, who were asked to provide continuous ratings of their emotional valence (Fig. 2B) during their first viewing using a slider-type affect rating dial, which enables dynamic assessment of emotional state using a computer mouse^30^. These data were used to test concordance with facial EMG changes. We used this cued-recall rating procedure for three reasons: (1) on-line ratings obtained by monitoring internal subjective states may interfere with natural physiological responses^31^; (2) a previous study reported strong positive correlations between on-line and recalled continuous ratings while participants viewed film clips^32^; and (3) we wanted to use the same procedures in both experiments (i.e., even while playing exercise games). In Experiment 2, we measured CS and ZM activity only using the wearable device while game-naïve participants played a Wii Bowling game to investigate more natural and subtle emotional experiences, including bodily movements (Fig. 1B). This game required participants to simulate actual bowling maneuvers with a motion-sensitive controller, which made using wired devices difficult. We expected that the game would induce moderate positive emotions, as reported in previous studies^33,34^. After finishing each frame, participants rated the overall subjective valence of the frame. After finishing the whole game, participants viewed their videotaped game displays so they could recall and continuously rate the emotional valence using a slider-type affect rating dial. We expected that CS and ZM activities recorded using the wearable device would consistently show negative and positive correlations, respectively, with continuous subjective valence experiences in both experiments.

## Results

### Experiment 1

**Figure 3:**
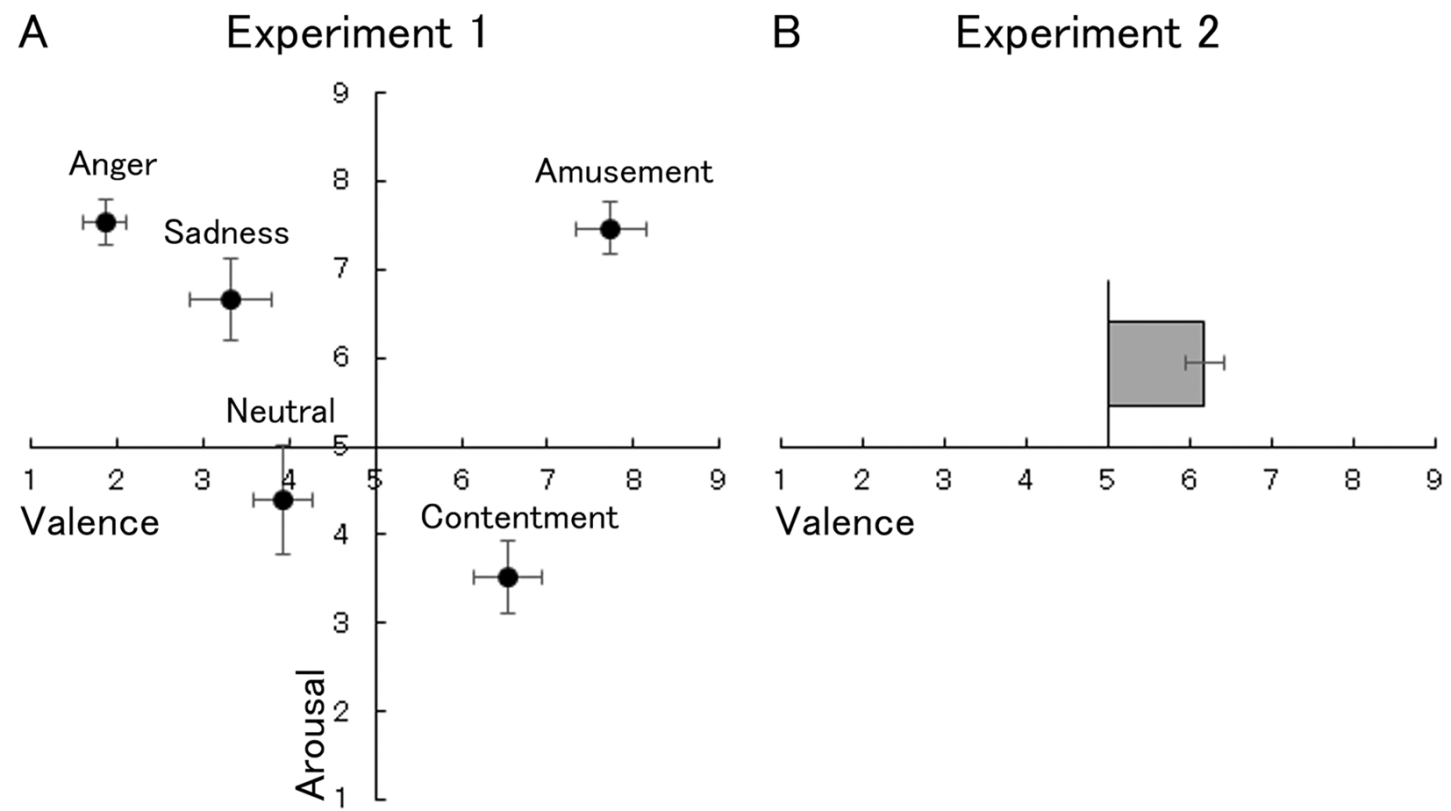
Mean (with *SE*) overall ratings in Experiments 1 (**A**) and 2 (**B**).

The overall ratings confirmed that the films elicited divergent valence experiences (Fig. 3A). Bonferroni-corrected one-sample *t*-tests (two-tailed) showed that all emotional films except for the neutral one induced valence and arousal states that were significantly different from the neutral state (*t*(14) > 3.55, corrected *p* < 0.05).

**Figure 4:**
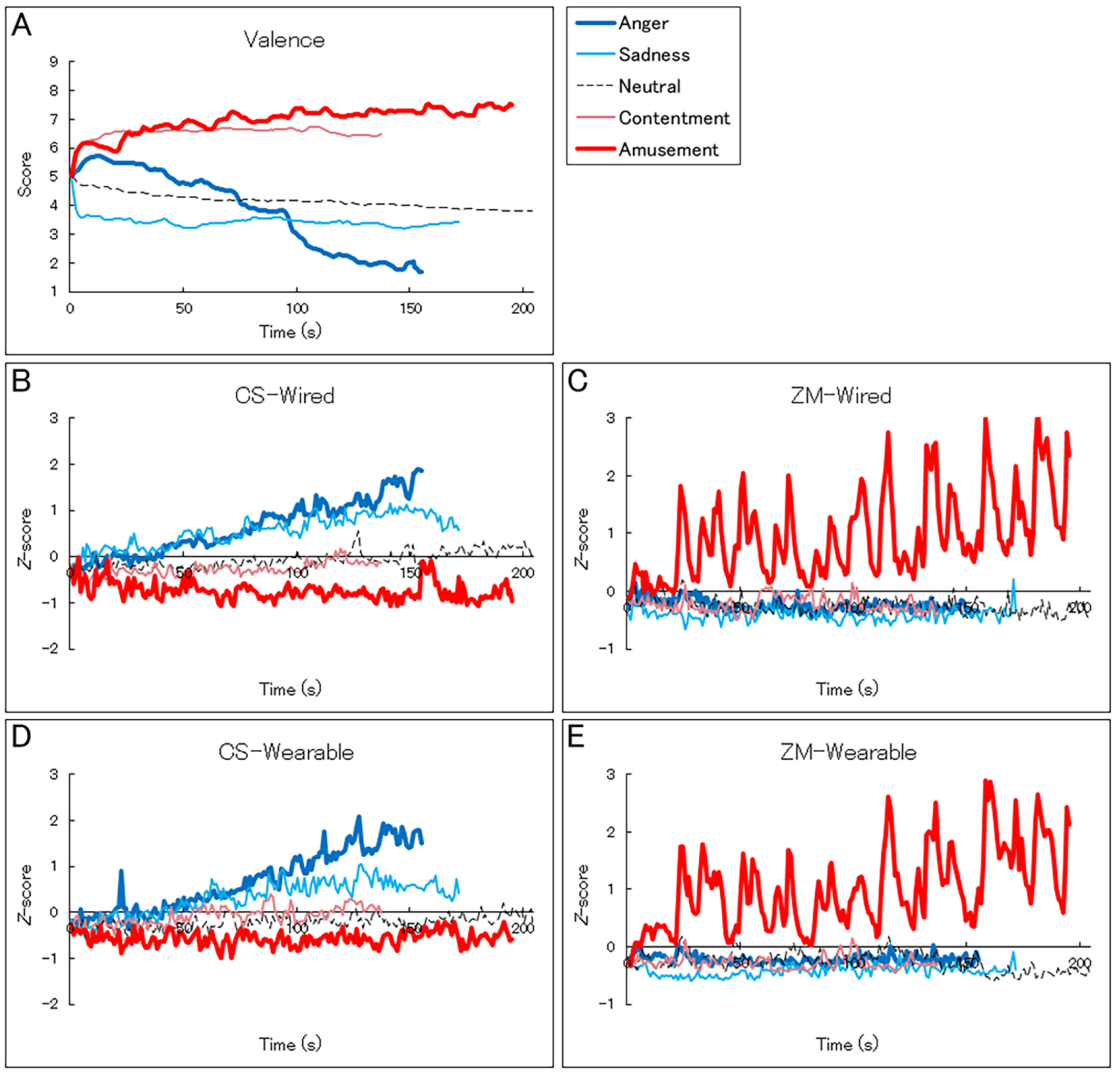
Group-mean second-by-second continuous subjective valence ratings (**A**) and facial electromyography (EMG) signals in Experiment 1. The facial EMG signals were recorded from the corrugator supercilii (CS) and zygomatic major (ZM) muscles using the wired (**B**,**C**) and wearable (**D**,**E**) devices. The facial EMG data were standardized within individuals.

Figure 4 shows the group-mean continuous subjective valence ratings and CS and ZM activities recorded using wired and wearable devices. The continuous subjective valence ratings showed that valence experiences changed during the films (e.g., the range of valence for anger films was 1.7–5.7). The waveform of CS and ZM activities according to both devices also showed changes during the films.

**Figure 5:**
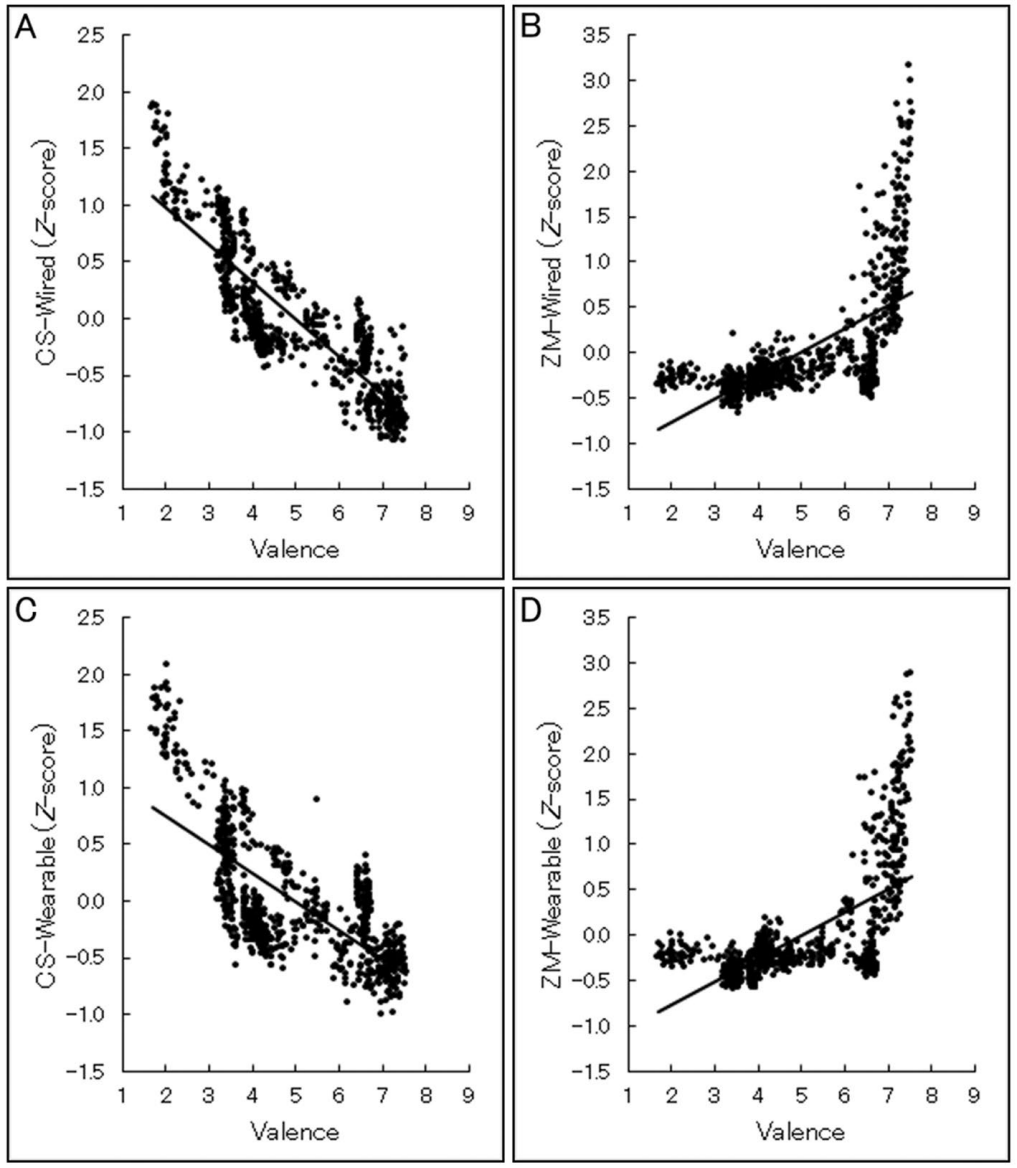
Group-averaged scatterplots and regression lines of relationships between continuous subjective valence ratings and facial electromyography (EMG) signals in Experiment 1. The facial EMG data were recorded from the corrugator supercilii (CS) and zygomatic major (ZM) muscles using the wired (**A**,**B**) and wearable (**C**,**D**) devices. The facial EMG data were standardized within individuals.

To visualize the concordance between subjective ratings and standardized facial EMG activities at the group level, we depicted the scatterplots and regression lines of the relationships using group-averaged data (Fig. 5). To statistically test concordance between subjective valence ratings and facial EMG data at the individual level, linear mixed effects models were constructed using subjective valence ratings and facial EMG data (after standardization) as the independent and dependent variables, respectively, with random by-participant slopes and intercepts^35^. The results revealed that all subjective valence–facial EMG relationships recorded using both wired and wearable devices were significant, including the positive and negative associations of the valence ratings with CS and ZM activities, respectively (*t*(14) > 3.78, *p* < 0.005; Table 1). Analysis of the standardized residuals revealed that the SDs of some were > 3 in all measures, indicating outliers (0.8, 2.2, 1.2, and 2.5% for CS-wired, ZM-wired, CS-wearable, and ZM- wearable, respectively). Therefore, the analyses were repeated after removing these data, and the results confirmed the same significant patterns as detected by the above analyses (*t*(14) > 2.66, *p* < 0.01).

**Table 1:** Results of multilevel modeling of relationships between continuous valence ratings and facial electromyography data in Experiment 1.

Statistic	Wired		Wearable	
	CS	ZM	CS	ZM
*Β*	**− 0.21**	**0.15**	**− 0.17**	**0.15**
*T*	**20.35**	**3.78**	**8.36**	**4.94**
*P*	**0.000**	**0.001**	**0.000**	**0.000**

There were 14 degrees of freedom, as calculated by the *m* – *l* – 1 approximation^46^.

*CS* corrugator supercilii, *ZM* zygomatic major.

Significant results (*p* < 0.05) are in bold.

### Experiment 2

The overall ratings showed that playing Wii Bowling induced positive valence states significantly different from the neutral state (one-sample *t*-test, *t*(21) = *p* < 0.001; Fig. 3B).

**Figure 6:**
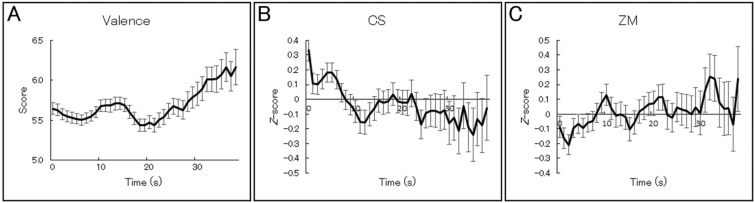
Grand-mean (with *SE*) second-by-second continuous subjective valence ratings (**A**) and facial electromyography data recorded from the corrugator supercilii (CS) (**B**) and zygomatic major (ZM) (**C**) muscles using the wearable device in Experiment 2. The facial EMG data were standardized within individuals.

Figure 6 shows the grand-mean second-by-second continuous subjective valence ratings and CS and ZM activities recorded with the wearable device. The waveforms for continuous subjective ratings showed that valence experiences followed a general positive trend with intermediate negative fluctuations. The CS and ZM activities showed negative and positive trends, respectively, with intermediate fluctuations.

**Figure 7:**
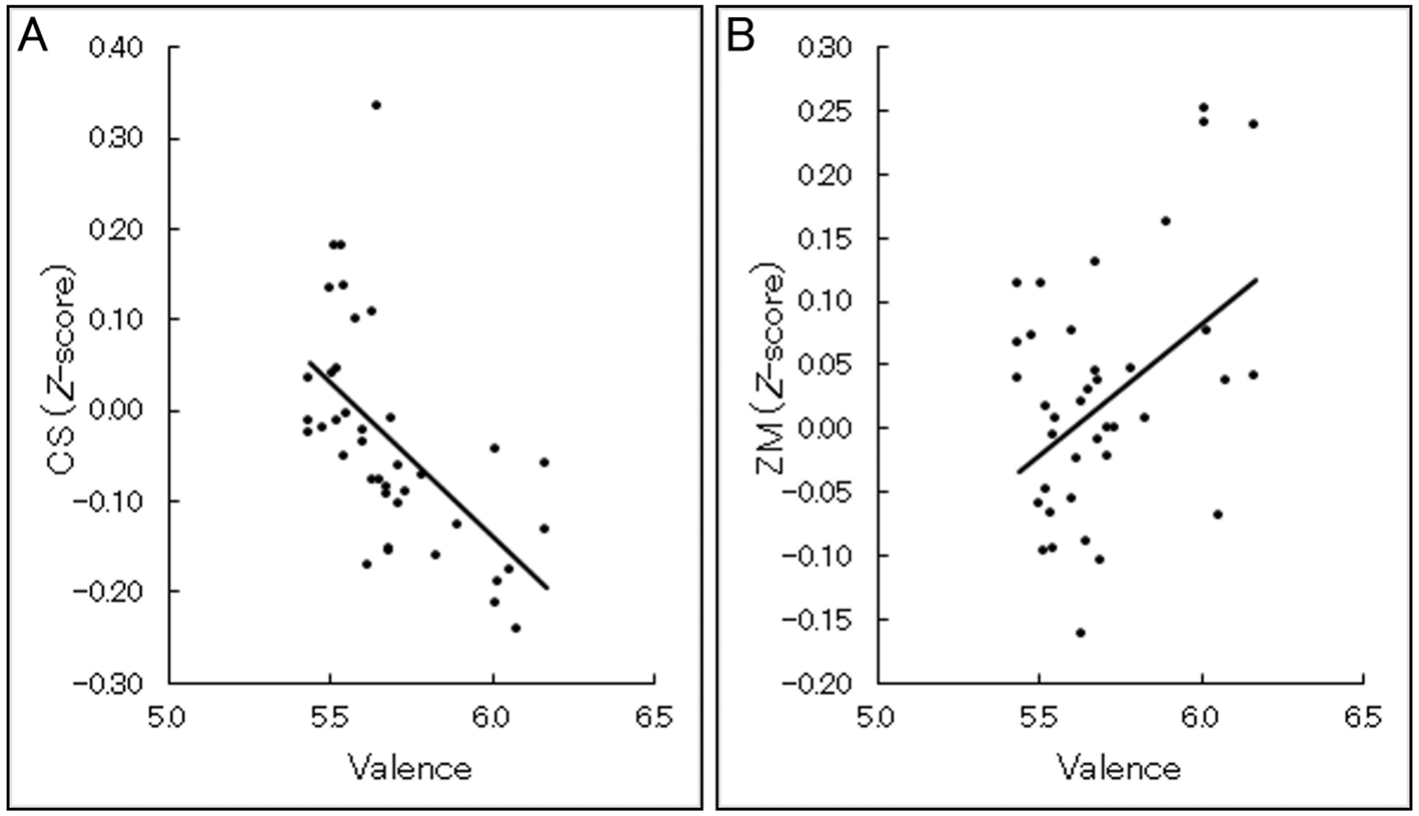
Group-averaged scatterplots and regression lines of relationships between continuous subjective valence ratings and facial electromyography (EMG) signals in Experiment 2. The facial EMG data were recorded from the corrugator supercilii (CS) (**A**) and zygomatic major (ZM) (**B**) muscles using the wearable device. The facial EMG data were standardized within individuals.

Figure 7 shows the group-averaged scatterplots and regression lines for relationships between continuous valence ratings and standardized facial EMG activities at the group level. Linear mixed effects models controlling for the random effects of participants and incorporating the same factors as the above analysis were constructed to test concordance at the individual level. The results showed significant associations with subjective valence ratings for both types of EMG data indicating negative and positive associations for CS and ZM activities, respectively (*t*(22) > 1.77, *p* < 0.05; Table 2). The standardized residuals included several data points > 3 SD according to both measures, suggesting outliers (1.4 and 2.1% for CS and ZM, respectively). After removing these data, we found that the associations with valence ratings remained marginally significant for both the CS and ZM data (*t*(22) > 1.32, *p* < 0.10).

**Table 2:** Results of multilevel modeling of relationships between continuous valence ratings and facial electromyography data in Experiment 2.

Statistic	CS	ZM
*β*	**− 0.03**	**0.03**
*t*	**1.77**	**2.13**
*p*	**0.046**	**0.022**

There were 22 degrees of freedom, as calculated by the *m* – *l* – 1 approximation^46^.

*CS* corrugator supercilii, *ZM* zygomatic major.

Significant results (*p* < 0.05) are in bold.

## Discussion

The results of the overall ratings in Experiment 1 showed that the emotional films induced valence and arousal states different from the neutral states in the expected direction. The overall ratings in Experiment 2 also showed more positive valence than the neutral state while playing on the Wii. These results are consistent with previous findings^28,33,34^ and indicate that our experimental settings reliably induced emotional states.

More importantly, the results of Experiment 1 showed concordance between participants’ subjective valence ratings and facial EMG activity while they viewed emotional films. These results are consistent with the findings of several previous studies that used wired devices and found concordance between continuous valence ratings and facial EMG activity^18–23,36–38^. This study newly demonstrates that facial EMG signals recorded using a wearable device can also be used to assess subjective emotional valence.

As in Experiment 1, the results of Experiment 2 revealed concordance between continuous subjective valence ratings and facial EMG activity while participants played Wii Bowling. Although the degree of concordance was weaker than that in Experiment 1, this difference may be due to weaker emotion elicitation and subtler changes in the course of emotions in Experiment 2 compared with Experiment 1. These results are consistent with the previous finding that wearable facial EMG devices allow recording of facial EMG signals related to emotional valence in naturalistic situations^27^. However, the previous study did not test for concordance between subjective emotional experiences and facial EMG signals. The results also corroborate previous studies that attempted to assess emotional experiences by recording facial muscle activities with wearable devices, although these studies did not record EMG data from the CS or ZM muscles^39–41^. To the best of our knowledge, our study provides the first evidence that a wearable facial EMG device can assess subjective emotional valence while participants move their bodies.

Our results have practical implications. They suggest that facial EMG signals recorded by a wearable device correspond to subjective emotional valence in naturalistic situations. Because emotional states influence many aspects of daily life^2^ and subjective ratings may be inappropriate or unavailable under some conditions^7^, it may be useful to be able to sense emotional valence states via facial EMG signals. For example, a previous study in consumer psychology reported that EDA recording in actual shopping situations allowed prediction of emotional states and buying behaviors^13^. A laboratory study using wired devices found that facial EMG data recorded from the ZM muscles predicted purchase decisions more sensitively than did EDA^42^. Together with these data, our data suggest that recording facial EMG in real shopping situations could provide predictive information related to customers’ emotional states and purchase behaviors, which could be useful in marketing research. In terms of clinical applications, a previous study reported that stress states could be predicted based on EDA data recorded using devices worn during daily life^14^. Another study using wired devices reported that facial EMG recorded from the CS muscles discriminated between stress and normal states more sensitively than did EDA signals^43^. Thus, facial EMG recording in everyday situations may provide valuable information regarding mental health states, and these data may complement clinical assessments based on interviews, behavior observation, and autonomic nervous system activity recording.

Several limitations of this study should be acknowledged. First, we analyzed only linear relationships between subjective valence and facial EMG activities. Previous studies reported several effective machine learning algorithms (e.g., support vector machines) for assessing emotional states based on physiological signals recorded using wearable devices^12^. Future investigation of such algorithms would increase the practical use of facial EMG signals recorded by wearable devices. Second, the arousal ratings may be related to the results of Experiment 1, where the overall ratings, including on the arousal scale, may have influenced the cued-recall valence ratings. In addition, given the quadratic relationship between valence and arousal ratings (Fig. 3A), the arousal dimension may be associated with facial EMG activity in a non-linear way. Future research assessing continuous arousal ratings, and exploring their relationships with facial EMG signals, may be helpful to clarify this issue. Finally, our experiments were conducted in the laboratory; so the emotion-sensing capacity of the wearable device in a naturalistic setting remains unproven. Further studies are therefore needed. In addition, it has been pointed out that ambulatory recording of physiological signals can be difficult due to multiple factors, including noise (e.g., the effect of sweating due to physical exertion) and less control of environmental factors^44^. Other technical problems, such as high variability in EMG signals during long-term recording and sensor misplacement, may occur in a naturalistic setting. Also, certain factors may limit the application of the wearable facial EMG device, such as stigmatization due to device appearance. Therefore, in addition to the new hardware reported in this study, software and application guidelines pertaining to the recording of facial EMG and emotion sensing in real life should be devised.

In conclusion, we aimed to determine whether the wearable device that we developed can reliably sense emotional valence, and conducted two laboratory experiments to this end. The results of Experiment 1 showed that the facial EMG signals recorded by both wired and wearable devices were associated with the subjective valence experiences reported by participants while viewing films. Similarly, the facial EMG signals recorded with the wearable device in Experiment 2 were also associated with the subjective valence experiences of participants, who were playing a game involving bodily movements. These data suggest that facial EMG signals recorded by a wearable device may reflect subjective emotional valence in naturalistic settings.

## Methods

### Experiment 1

#### Participants

Fifteen Japanese volunteers (eight females; mean ± SD age, 21.6 ± 2.4 years) participated in this experiment. The required sample size was determined using an a priori power analysis using G*Power software ver. 3.1.9.2^45^ based on a preliminary experiment with a different sample (*n* = 10) using similar film stimuli and EMG recording. As an approximation of the present analysis of subjective–physiological concordance, we followed a two-step procedure^35^ that included estimating separate correlation coefficients for each participant and conducting *t*-tests to determine whether the mean coefficient across participants could be significantly different from zero. The effect size was estimated from the results, assuming an *α* level of 0.05 and a power (1 – *β*) of 0.80. The results of the power analysis showed that more than 12 participants were needed. All participants had normal or corrected-to-normal visual acuity. After a detailed explanation of the experimental procedure, all participants provided informed consent. Our study was approved by the Ethics Committee of the Unit for Advanced Studies of the Human Mind, Kyoto University. The experiment was conducted in accordance with institutional ethical provisions and the Declaration of Helsinki.

#### Apparatus

Experimental events were controlled by Presentation software (Neurobehavioral Systems, Berkeley, CA, USA) implemented on Windows computers (HP Z200 SFF, Hewlett–Packard Japan, Tokyo, Japan). The stimuli were presented on a 19-inch cathode ray tube monitor (HM903D-A, Iiyama, Tokyo, Japan) with a refresh rate of 100 Hz and a resolution of 1024 × 768 pixels. Another Windows laptop computer (CF-SV8, Panasonic, Tokyo, Japan) was used for the continuous rating.

#### Stimuli

We used the three film stimuli developed by Gross and Levenson^46^. The stimulus scenes were from the following films: “Cry Freedom,” “The Champ,” and “Abstract Shapes,” representing highly negative (anger), moderately negative (sadness), and neutral stimuli, respectively. The ability of the stimuli to elicit the target emotions effectively was validated in a Japanese sample^28^. We replaced Gross and Levenson’s^46^ moderately positive (contentment) and highly positive (amusement) films with conceptually similar films of a beach scene and a humorous dialogue, which were selected from commercial films (Maldives Nature, Japan Media Supply, Tokyo, Japan; M-1 Grand Prix The Best 2007–2009, Yoshimoto, Tokyo, Japan), because the original films were of low resolution and not in Japanese, respectively. The mean ± SD time of the film stimuli was 173.4 ± 27.7 s (range: 138–205 s). One film from Gross and Levenson^46^ depicting a scene from “Silence of the Lambs” was used for practice. The stimuli were presented at 640 horizontal × 480 vertical pixels, subtending a visual angle of about 25.5° horizontal × 11° vertical.

#### Procedure

The experiment was conducted individually in an electrically shielded soundproof room (Science Cabin, Takahashi Kensetsu, Tokyo, Japan). Upon arrival, participants were told that the experiment concerned sweat gland activity while evaluating film clips, which was the cover story used to conceal the purpose of the study regarding facial EMG recording. Participants were given about 10 min to adapt to the experimental room. First, one film was presented for practice. Then, five film stimuli were presented. We pseudorandomized the film presentation order across participants so that no two films with the same valence dimension were presented in succession.

For each trial, the film stimulus was presented after a fixation point (a small white “ + ” on a black background) and a 10-s period of plain white. Then, after the 10-s white screen, the affect grid^29^, which graphically assesses the two dimensions of valence and arousal on nine-point scales, was presented, and participants used a keyboard to rate their overall subjective emotional experience while viewing the film. A black screen, which was controlled to vary randomly from 24 to 30 s in duration to reduce psychological expectation and periodic physiological activity, was presented during the inter-trial interval. Physiological data were continuously recorded for all trials.

Following completion of all trials, participants viewed the stimuli again and were asked to recall and rate the valence associated with each clip during the first viewing using a slider-type affect rating dial^30^. Horizontal nine-point scales were presented, participants responded using a mouse, and the coordinates of the mouse were recorded.

To confirm that the cued-recall continuous rating procedure could provide data positively correlated with on-line continuous ratings, as reported in a previous study^32^, a preliminary experiment was conducted with a different sample (*n* = 15; 8 females; mean ± SD age, 21.5 ± 1.6 years). The films were presented in the same way as in the main experiment for two different tasks. In the first on-line rating task, participants were instructed to rate on-line their emotional valence while watching the films. In the second cued-recall rating task, the participants received the same instructions as in the main experiment and recalled the emotional valence during the first viewing. Pearson’s product-moment correlation coefficients between on-line and cued-recall ratings were calculated for each film clip for each participant and analyzed using Bonferroni-corrected one-sample *t*-tests (two-tailed). The results showed significant positive correlations for all film clips (mean ± SD* r* = 0.92 ± 0.01, 0.59 ± 0.09, 0.58 ± 0.07, 0.57 ± 0.09, and 0.58 ± 0.09 for anger, sadness, neutral, contentment, and amusement, respectively; *t*(14) > 6.35, Bonferroni-corrected *p* < 0.001). The results suggested that cued-recalled and on-line valence ratings are likely to be similar.

#### Facial EMG recording

EMG data were recorded from the CS and ZM muscles with a wired device using sets of pre-gelled, self-adhesive 0.7-cm Ag/AgCl electrodes with 1.5-cm inter-electrode spacing (Prokidai, Soraku-gun, Japan). The electrodes were placed according to guidelines^47,48^. A ground electrode was placed on the forehead. The data were amplified, filtered online (band pass: 20–400 Hz), and sampled at 1,000 Hz using an EMG-025 amplifier (Harada Electronic Industry, Sapporo, Japan) and the PowerLab 16/35 data acquisition system with LabChart Pro v8.0 software (ADInstruments, Dunedin, New Zealand). A 20 Hz low-cut filter was applied because this has been shown to effectively remove artifacts related to hand and leg movements from facial EMG data^49^. A video recording was made unobtrusively using a digital web camera (HD1080P, Logicool, Tokyo, Japan) to check for motion artifacts and facial positions. Although the skin potential level and heart rate were additionally recorded using the same system, we do not report these data.

In terms of facial EMG recording using a wearable device, we developed a compact device that can be worn as eyeglasses (Fig. 1A). The width, depth, and height of the device were 67, 53, and 13 mm, respectively. ADS1298R (Texas Instruments Inc., Dallas, TX, USA) was used for the facial EMG measurements. ADS1298R is an analog front-end (AFE) circuit that includes filters, amplifiers, and an analog-to-digital converter (ADC) for measuring bio-potential signals, such as EMG and electrocardiography. The specification and parameters of the ADS1298R AFE are as follows: input referred noise, 4 μVpp; common mode rejection ratio, − 115 dB; ADC, 8-channel 24-bit; and programmable gain, 6. This device sampled the EMG data of the CS and ZM muscles at 500 Hz using the same electrodes as the wired device. The measured data were transmitted to a recording terminal by Bluetooth low-energy transmission. The recorded data were filtered offline (low cut: 20 Hz) and then the 51-point moving average was subtracted to remove baseline drift.

The electrodes of the wired and wearable devices were placed on opposite sides of each participant’s face. The sides were counterbalanced across participants.

#### Data analysis

Overall ratings were analyzed using one-sample *t*-tests to identify significant differences from the neutral state (i.e., 5). *P*-values were two-tailed and Bonferroni-corrected for multiple testing (i.e., divided by 10).

EMG data analyses were performed using Psychophysiological Analysis software 3.3 (Computational Neuroscience Laboratory of the Salk Institute, San Diego, CA, USA), the machine learning toolbox, and in-house programs implemented in MATLAB 2018 (MathWorks, Natick, MA, USA). For preprocessing, all EMG data recorded using the wired and wearable devices were sampled for 10 s during the pre-stimulus baseline period (while a white screen was presented) and then as the film was presented during each trial. One of the authors blindly checked the video data and confirmed that participants did not cause large motion artifacts. For each trial, the data were rectified, baseline-corrected with respect to the mean value over the pre-stimulus period and averaged in intervals of 1000 ms. Data from all film conditions were concatenated and then standardized within individuals.

To statistically evaluate the individual-level concordance between subjective valence ratings and facial EMG activity, we constructed linear mixed effects models using continuous subjective ratings and facial EMG data as the independent and dependent variables, respectively, and the random by-participant slopes and intercepts^35^. A maximum likelihood estimate was calculated. The beta estimates of the subjective ratings were evaluated using *t*-tests (one tailed), with the degrees of freedom calculated using the *m* – *l* – 1 approximation, which was validated through Monte Carlo simulations^50^. Additionally, standardized residuals were calculated to check for outliers. To illustrate group-level concordance between subjective valence ratings and facial EMG activity, we created scatterplots and regression lines of the above relationships using group-mean data (Fig. 3). A previous study examining the concordance between continuous subjective valence ratings and facial EMG activity across time in response to emotional films found that the cross-correlation at zero lag was the highest, and Pearson’s correlation coefficients successfully revealed the concordance^38^. Hence, although some previous studies calculated cross-correlations^36^, we analyzed the data at a lag of zero. Because the analysis of the standardized residuals revealed outliers at > 3 SD, the same models were constructed after removing outliers. The results were considered significant at *p* < 0.05.

We conducted preliminary analyses regarding the effect of sex by constructing the above models and adding the covariate of sex. We found that all reported significant results remained significant, and there was no significant effect of sex (*p* > 0.10). Based on these null findings, this factor was disregarded.

### Experiment 2

#### Participants

Data from 23 Japanese volunteers (14 females; mean ± SD age, 22.0 ± 1.7 years) were recorded and analyzed. Participants who did not frequently play Wii Bowling were recruited through advertising at Kyoto University. Although two additional participants actually participated in the experiment, their data were not recorded due to equipment errors. As in Experiment 1, the required sample size was determined using an a priori power analysis. Data from a preliminary experiment with a different sample (*n* = 10) using a similar free movement paradigm and the wearable EMG device were used. The power analysis showed that more than 21 participants were needed. All participants had normal or corrected-to-normal visual acuity. After a detailed explanation of the experimental procedure, all participants provided informed consent. Our study was approved by the Ethics Committee of the Unit for Advanced Studies of the Human Mind, Kyoto University. The experiment was performed in accordance with institutional ethical provisions and the Declaration of Helsinki.

#### Apparatus

A Nintendo Wii hardware and Wii Sports software (Nintendo, Kyoto, Japan) were used for Wii Bowling. The game display was presented on a 20-inch liquid–crystal display (2007FPb, Dell, Round Rock, TX, USA). A laptop computer running Windows (CF-SV8, Panasonic, Tokyo, Japan) was used for ratings.

#### Procedure

The open field experiment was conducted individually in the laboratory. Upon arrival, participants were told that the experiment concerned sweat gland activity while playing Wii Bowling, which was the cover story used to conceal the purpose of the study. Participants were given about 10 min to adapt to the experimental room. First, three frames were played for practice. Then, participants were instructed to play for 10 min.

During the game play, participants played Wii Bowling, which simulates actual bowling in three-dimensional cyber space. Wii Bowling was selected because it was reported to be the most popular and the easiest to learn of the Wii sports games^51^. Participants performed the bodily motions associated with actual bowling with a motion-sensitive controller. In addition, they manipulated buttons on the controller to release balls at the appropriate time. Figure 1B shows an illustration of Experiment 2 with an amateur model who submitted written consent to show her face in scientific journals. In each frame, participants threw the ball once (if they scored a strike), twice, or three times (only for the last game with strikes or spares). Participants were instructed to observe a replay of each frame after each frame. Participants then rated the valence of their experience during the frame using a horizontal nine-point scale presented on a laptop computer. The mean ± SD frame length was 26.7 ± 8.9 s, and the mean ± SD number of throws per frame was 1.9 ± 0.1.

After play concluded, replays of the game were presented while participants were asked to recall and provide continuous ratings of their valence experiences using a nine-point slider-type affect rating dial^30^. Participants responded using a mouse, and the coordinates of the mouse were recorded.

#### Facial EMG recording

Facial EMG data from participants’ left-side CS and ZM muscles were recorded using the same wearable device used in Experiment 1.

#### Data analysis

Preprocessing for EMG data was conducted in the same way as in Experiment 1 except that we did not create baseline periods, we removed some artifact trials, and we did not perform baseline correction. The EMG data were recorded during the whole period (i.e., from the start to the end of the replay) for each frame. Because we did not control participants’ behaviors during games, there were no clear baseline periods. One of the authors blindly checked the video data and removed as artifacts the data from several time points during which participants touched the glasses/wearable devices, made bodily movements that moved the wearable devices, or made mouth movements related to muttering (total, 6.5%). For each frame, the data were rectified and averaged in intervals of 1000 ms without baseline corrections.

Statistical analyses were also identical to those performed in Experiment 1. To depict the grand-mean second-by-second ratings and facial EMG data, we calculated means and SEs across all frames for all participants. The end point (i.e., 39 s) for figures was determined to cover 90% of the total frames.

## References

[1] Lyubomirsky S (2001). Why are some people happier than others? The role of cognitive and motivational processes in well-being. Am. Psychol..

[2] Meiselman HL (2015). A review of the current state of emotion research in product development. Food Res. Int..

[3] Ekman, P. Basic emotions in *The Handbook of Cognition and Emotion*. (eds. Dalgleish, T. & Power, T.) 45–60 (Sussex, John Wiley & Sons, 1999).

[4] Russell JA (2003). Core affect and the psychological construction of emotion. Psychol. Rev..

[5] Yang ZY, He LY (2011). Goal, customer experience and purchase intention in a retail context in China: An empirical study. Afr. J. Bus. Manag..

[6] Wichers M (2007). Genetic risk of depression and stress-induced negative affect in daily life. Br. J. Psychiatry.

[7] Li S, Scott N, Walters G (2015). Current and potential methods for measuring emotion in tourism experiences: a review. Curr. Issues Tour..

[8] Trull TJ, Ebner-Priemer U (2013). Ambulatory assessment. Annu. Rev. Clin. Psychol..

[9] Peake JM, Kerr G, Sullivan JP (2018). A critical review of consumer wearables, mobile applications, and equipment for providing biofeedback, monitoring stress, and sleep in physically active populations. Front. Physiol..

[10] Zanstra YJ, Johnston DW (2011). Cardiovascular reactivity in real life settings: measurement, mechanisms and meaning. Biol. Psychol..

[11] Malhi GS (2017). The promise of digital mood tracking technologies: are we heading on the right track?. Evid. Based Ment. Health.

[12] Smets E, De Raedt W, Van Hoof C (2019). Into the Wild: The challenges of physiological stress detection in laboratory and ambulatory settings. IEEE J. Biomed. Health. Inform..

[13] Groeppel-Klein A (2005). Arousal and consumer in-store behavior. Brain Res. Bull..

[14] Can YS, Chalabianloo N, Ekiz D, Ersoy C (2019). Continuous stress detection using wearable sensors in real life: Algorithmic programming contest case study. Sensors.

[15] Cacioppo, J. T., Berntson, G. G. & Klein, D. J. What is an emotion? The role of somatovisceral afference, with special emphasis on somatovisceral "illusions" in *Emotion and Social Behavior. ix*. (ed. Clark, M. S.) 63–98 (Thousand Oaks, Sage, 1992).

[16] Lang PJ, Bradley MM, Cuthbert BN (1998). Emotion, motivation, and anxiety: Brain mechanisms and psychophysiology. Biol. Psychiatry.

[17] Greenwald MK, Cook EW, Lang PJ (1989). Affective judgment and psychophysiological response: Dimensional covariation in the evaluation of pictorial stimuli. J. Psychophysiol..

[18] Lang PJ, Greenwald MK, Bradley MM, Hamm AO (1993). Looking at pictures: Affective, facial, visceral, and behavioral reactions. Psychophysiology.

[19] Larsen JT, Norris CJ, Cacioppo JT (2003). Effects of positive and negative affect on electromyographic activity over zygomaticus major and corrugator supercilii. Psychophysiology.

[20] Tan JW (2012). Repeatability of facial electromyography (EMG) activity over corrugator supercilii and zygomaticus major on differentiating various emotions. J. Ambient. Intell. Humaniz. Comput..

[21] Sato W, Fujimura T, Kochiyama T, Suzuki N (2013). Relationships among facial mimicry, emotional experience, and emotion recognition. PLoS ONE.

[22] Sato W, Kochiyama T, Yoshikawa S (2020). Physiological correlates of subjective emotional valence and arousal dynamics while viewing films. Biol. Psychol..

[23] Bradley MM, Lang PJ (2000). Affective reactions to acoustic stimuli. Psychophysiology.

[24] Kehri, V., Patil, S. S. & Awale, R. N. Analysis of facial EMG signal for emotion recognition using wavelet packet transform and SVM in *Machine intelligence and signal analysis* (eds. Tanveer, M. & Pachori, R B.) 247–257 (Singapore, Springer, 2018).

[25] Kunecke J, Hildebrandt A, Recio G, Sommer W, Wilhelm O (2014). Facial EMG responses to emotional expressions are related to emotion perception ability. PLoS ONE.

[26] 't Hart, B., Struiksma, M. E., van Boxtel, A. & van Berkum, J. J. A. Emotion in stories: Facial EMG evidence for both mental simulation and moral evaluation. *Front. Psychol*. **9**, 613 (2018). 10.3389/fpsyg.2018.00613 PMC5937160 29760671

[27] Inzelberg L, Rand D, Steinberg S, David-Pur M, Hanein Y (2018). A wearable high-resolution facial electromyography for long term recordings in freely behaving humans. Sci. Rep..

[28] Sato W, Noguchi M, Yoshikawa S (2007). Emotion elicitation effect of films in a Japanese sample. Soc. Behav. Pers..

[29] Russell JA, Weiss A, Mendelsohn GA (1989). Affect grid: A single-item scale of pleasure and arousal. J. Pers. Soc. Psychol..

[30] Ruef, A. M. & Levenson, R. W. Continuous measurement of emotion: The affect rating dial in *Handbook of Emotion Elicitation and Assessment*. (eds. Coan, J. A. & Allen, J. J. B.) 286–297 (Oxford University Press, 2007).

[31] Rosenberg EL, Ekman P (1994). Coherence between expressive and experiential systems in emotion. Cogn. Emot..

[32] Mauss IB, Levenson RW, McCarter L, Wilhelm FH, Gross JJ (2005). The tie that binds? Coherence among emotion experience, behavior, and physiology. Emotion.

[33] Hsu JK (2011). A "Wii" bit of fun: the effects of adding Nintendo Wii Bowling to a standard exercise regimen for residents of long-term care with upper extremity dysfunction. Physiother. Theory Pract..

[34] Rosenberg D (2010). Exergames for subsyndromal depression in older adults: A pilot study of a novel intervention. Am. J. Geriatr. Psychiatry.

[35] Richter T (2006). What is wrong with ANOVA and multiple regression? Analyzing sentence reading times with hierarchical linear models. Discourse Process..

[36] Butler EA, Gross JJ, Barnard K (2014). Testing the effects of suppression and reappraisal on emotional concordance using a multivariate multilevel model. Biol. Psychol..

[37] Dan-Glauser ES, Gross JJ (2013). Emotion regulation and emotion coherence: evidence for strategy-specific effects. Emotion.

[38] Golland Y, Hakim A, Aloni T, Schaefer S, Levit-Binnun N (2018). Affect dynamics of facial EMG during continuous emotional experiences. Biol. Psychol..

[39] Gruebler, A. & Suzuki, K. A wearable interface for reading facial expressions based on bioelectrical signals. *Proc. Int. Conf. Kansei Eng. Emotional Res.* **2010**, 1–10 (2010).

[40] Kwon J, Kim DH, Park W, Kim L (2016). A wearable device for emotional recognition using facial expression and physiological response. Conf. Proc. IEEE Eng. Med. Biol. Soc..

[41] Scheirer, J., Fernandez, R. & Picard, R. W. Expression Glasses: A wearable device for facial expression recognition. *Proc. SIGCHI Conf. Hum. Factor. Comput. Syst*. 262–263 (1999).

[42] Somervuori O, Ravaja N (2013). Purchase behavior and psychophysiological responses to different price levels. Psychol. Mark..

[43] Shapiro MS, Rylant R, de Lima A, Vidaurri A, van de Werfhorst H (2017). Playing a rigged game: Inequality’s effect on physiological stress responses. Physiol. Behav..

[44] Birenboim A, Dijst M, Scheepers FE, Poelman MP, Helbich M (2019). Wearables and location tracking technologies for mental-state sensing in outdoor environments. Prof. Geogr..

[45] Faul F, Erdfelder E, Lang AG, Buchner A (2007). G*Power 3: A flexible statistical power analysis program for the social, behavioral, and biomedical sciences. Behav. Res. Methods.

[46] Gross JJ, Levenson RW (1995). Emotion elicitation using films. Cogn. Emot..

[47] Fridlund AJ, Cacioppo JT (1986). Guidelines for human electromyographic research. Psychophysiology.

[48] Schumann NP, Bongers K, Guntinas-Lichius O, Scholle HC (2010). Facial muscle activation patterns in healthy male humans: a multi-channel surface EMG study. J. Neurosci. Methods.

[49] Van Boxtel A (2001). Optimal signal bandwidth for the recording of surface EMG activity of facial, jaw, oral, and neck muscles. Psychophysiology.

[50] Elff, M., Heisig, J. P., Schaeffer, M. & Shikano, S. No need to turn Bayesian in multilevel analysis with few clusters: How frequentist methods provide unbiased estimates and accurate inference. *SocArXiv*, **2016**, December 10 (2016).

[51] Cyarto E, Kuys SS, Henwood TR, Blackberry I (2011). Can Wii work it out?. Telecommun. J. Aust..

